# Energetic costs regulated by cell mechanics and confinement are predictive of migration path during decision-making

**DOI:** 10.1038/s41467-019-12155-z

**Published:** 2019-09-13

**Authors:** Matthew R. Zanotelli, Aniqua Rahman-Zaman, Jacob A. VanderBurgh, Paul V. Taufalele, Aadhar Jain, David Erickson, Francois Bordeleau, Cynthia A. Reinhart-King

**Affiliations:** 1 Nancy E. and Peter C. Meinig School of Biomedical Engineering, Cornell University, Ithaca, NY 14853 USA; 2 Department of Biomedical Engineering, Vanderbilt University, Nashville, TN 37235 USA; 3 Sibley School of Mechanical and Aerospace Engineering, Cornell University, Ithaca, NY 14853 USA; 4 CHU de Québec-Université Laval Research Center (Oncology division), Université Laval Cancer Research Center and Faculty of Medicine, Université Laval, Québec, QC G1R 3S3 Canada

**Keywords:** Extracellular matrix, Biomedical engineering

## Abstract

Cell migration during the invasion-metastasis cascade requires cancer cells to navigate a spatially complex microenvironment that presents directional choices to migrating cells. Here, we investigate cellular energetics during migration decision-making in confined spaces. Theoretical and experimental data show that energetic costs for migration through confined spaces are mediated by a balance between cell and matrix compliance as well as the degree of spatial confinement to direct decision-making. Energetic costs, driven by the cellular work needed to generate force for matrix displacement, increase with increasing cell stiffness, matrix stiffness, and degree of spatial confinement, limiting migration. By assessing energetic costs between possible migration paths, we can predict the probability of migration choice. Our findings indicate that motility in confined spaces imposes high energetic demands on migrating cells, and cells migrate in the direction of least confinement to minimize energetic costs. Therefore, therapeutically targeting metabolism may limit cancer cell migration and metastasis.

## Abstract (web summary)

Migrating cells tune their energy utilization in response to their microenvironment, but how cellular energetics direct navigation remains unclear. Here, the authors report that energetic costs for motility, regulated by cell mechanics and confinement, predict the probability of migration choice.

## Introduction

Cell migration is a critical aspect of the invasion-metastasis cascade and is significantly influenced by the microenvironment. The physical properties of the extracellular matrix (ECM) have been identified as key mediators of cell behavior and determine requirements for motility^1–3^. During cancer progression, the ECM commonly becomes deregulated and disorganized^4^ resulting in a highly heterogeneous ECM containing restricting pores, cross-sectional areas, and channel-like tracks^5^. These tight interstitial spaces can range from ~3 to 30 μm in width^2^, creating complex topographies that present directional choices to migrating cells^5–7^. Notably, channel-like tracks in the matrix, which are native to the ECM or prepatterned by cells themselves using metalloproteinases (MMPs), provide physical guidance, and offer a path of least resistance for migrating cells^2,8,9^. Once channel-like tracks are created by “leading” cancer cells, other “following” cancer cells utilize these microtracks to rapidly disseminate in an unimpeded, MMP-independent manner^10^. This mode of migration may explain the limited clinical success of MMP inhibitors to treat metastasis^11^. As these microtracks in the matrix provide strong proinvasive cues to tumor cells, understanding the mechanisms of cancer cell motility through physiologically relevant confining tracks will be critical to developing therapeutic strategies to target metastasis.

To navigate these physical barriers and migrate, cells dynamically coordinate cellular machinery to generate forces and remodel their cytoskeleton and/or the surrounding matrix^12–14^, both of which are energy-demanding processes^15–17^. Cells generally meet such energy needs through the dephosphorylation of ATP into ADP. Maintaining an adequate supply of ATP is critical for cellular remodeling^18^, and ATP production is determined by fluctuating energetic demands of the cell^19,20^. Our recent work indicates that individual migrating cells tune their energy utilization relative to the structure and mechanics of their microenvironment^21^, and collectively migrating cells employ relay-like behavior to invade through physically challenging and energy-demanding environments^22^. However, the role of cellular energetics in directional decision-making during migration through spatially complex microenvironments is not well understood.

Here, we show that when presented with migration choices of varying confinement, MDA-MB-231 cells preferentially migrate in the direction of least confinement to minimize energetic costs. Using a computational model and in vitro experiments, we demonstrate that energetic costs for migration through confined spaces are mediated by a balance between cell and matrix compliance and the degree of spatial confinement to direct migration decision-making. Increased cell stiffness limits cell body deformation and requires cell-induced matrix displacement for migration through narrow spaces. The cellular work required for matrix displacement drives the energy requirements for migration, and these energetic costs exponentially increase with increasing cell stiffness. At high degrees of spatial confinement as well as high cell stiffness and/or high matrix stiffness, elevated energetic costs for movement restrict migration into narrower confined spaces. Using this framework, we can accurately predict the probability of migration decisions by calculating the energetic costs between possible migration paths. Together, these findings provide insight into the role of cellular energetics in migration and demonstrate that energetic costs, in part, determine a cell’s ability to navigate complex environments.

## Results

### Cells sense path size during migration decision-making

**Fig. 1:**
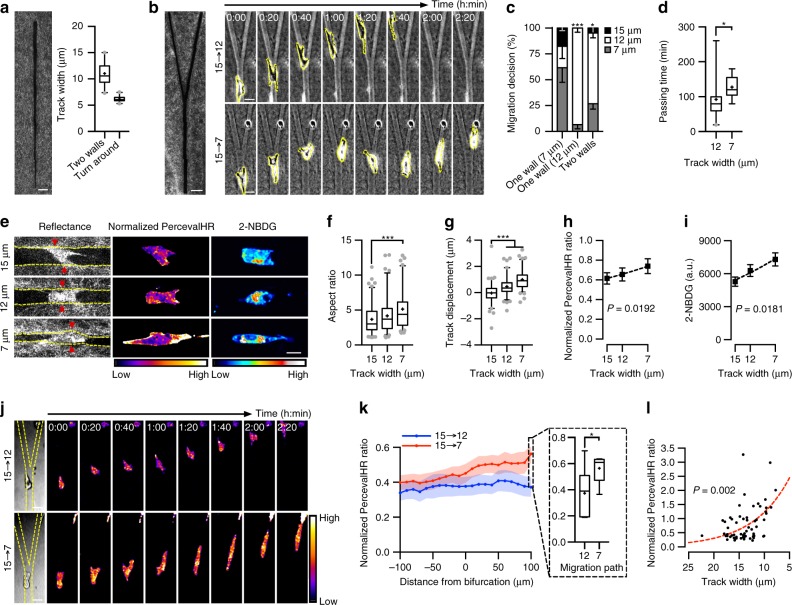
Spatial confinement influences migration decision-making, cellular ATP:ADP ratio, and glucose uptake. **a** Confocal reflectance image of a tapered collagen microtrack, and track width when cells contact two side walls of the microtrack or turn around and reverse migration direction (*n* = 20 cells). **b** Confocal reflectance image of a Y-shaped collagen microtrack and time-series images of decision-making during migration in Y-shaped microtracks (cell body outlined in yellow). **c** Final migration choice of cells based on cell-matrix contact before reaching the bifurcation (*n* = 34, 29, 41 cells for one wall (7 μm), one wall (12 μm), or two walls). **d** Time taken for cells touching two walls to pass through the bifurcation and enter a branch (*n* = 29 cells). **e** Confocal reflectance images of microtrack structure, and normalized PercevalHR ratio and 2-NBDG heatmaps of cells in each microtrack (yellow lines show microtrack walls; red arrowheads show areas of matrix displacement around the cell body). **f**, **g** Quantification of cell body aspect ratio (**f**) and track displacement (**g**) for cells in 15, 12, and 7 μm microtracks (*n* = 95, 96, and 91 cells, respectively). **h**, **i** Normalized PercevalHR ratio (*n* = 95, 96, and 91 cells, respectively) (**h**) and 2-NBDG uptake (*n* = 54, 40, and 56 cells, respectively) (**i**) for cells in 15, 12, and 7 μm microtracks. **j** Intracellular ATP:ADP ratio (normalized PercevalHR ratio) during decision-making in Y-shaped microtracks (yellow lines show microtrack walls). **k** Quantification of normalized PercevalHR ratio as a function of distance from the bifurcation and after final migration choice (*n* = 10 cells for 12 μm path, 5 cells for 7 μm path). **l** Normalized PercevalHR ratio in tapered collagen microtracks (*n* = 62 cells). Data shown as median ± interquartile range (box), 5th–95th percentiles (whiskers), and mean (+) (**a**, **d**, **f**, **g**, **k**), or mean ± s.e.m. (**c**, **h**, **i**); dashed lines show exponential growth; Clopper–Pearson confidence interval for observed proportion (**c**), two-tailed Mann–Whitney test (**d**, **f**, **g**, **k**), or extra sum-of-squares F-test (**h**, **i**, **l**); *P < 0.05, **P < 0.01, ***P < 0.001. Scale bar, 50 μm (**a**, **b**), 25 μm (**b**, **j**), and 15 μm (**e**)

To recreate directional choices presented to cancer cells during migration, we utilized microfabrication to create Y-shaped microtracks. Microfabrication enables the creation of well-defined channels to study migration; however, most channels are molded into polydimethylsiloxane (PDMS)^23–26^. Here, we created three-dimensional collagen microtracks^27^ thereby mimicking the complex architecture of the native peritumoral ECM and allowing for mechanoreciprocity between cells and the matrix, a key determinant for migration^1^. To determine relevant physical dimensions for the bifurcations of the Y-shaped microtrack, we first created tapered collagen microtracks with widths decreasing from 20 to 5 μm and assessed spatial confinement, cell-matrix contact, and cell motility (Fig. 1a). MDA-MB-231 cells became fully confined, contacting two side walls of the microtrack at a track width of 11.020 ± 0.471 μm (mean ± s.e.m.) and cells reversed their migration direction at a track width of 6.212 ± 0.126 μm (mean ± s.e.m.), consistent with MDA-MB-231 cell body diameter^28^ and nucleus diameter^29^, as well as the physical limit of MMP-independent migration^30^. Based on these dimensions, we created a Y-shaped collagen microtrack consisting of a 15 μm feeder track bifurcating into 12 and 7 μm wide branches to study migration decision-making (Fig. 1b). Consistent with previous observations^25^, contact guidance determined migration path when cells contacted a single side wall of the 15 μm feeder track. However, when contacting both side walls of the feeder track, cells preferentially and more readily migrated into the wider path (~70%) with a faster passage into the wider branch (Fig. 1c, d). When moving into the narrower path, slower passage time was also accompanied by increased probing at the bifurcation (Supplementary Movies 1 and 2). These data indicate that cells actively probe their surrounding matrix to sense path size in choosing a migration direction, and preferentially migrate along the path of least resistance.

Migration into confined spaces requires cells to either remodel their cytoskeleton or deform the surrounding matrix^12–14^. During migration through Y-shaped collagen microtracks, we found cells altered their morphology and deformed the side walls in more confined tracks, a finding unique to our collagen microtracks compared with traditional PDMS channels^23–25^. As spatial confinement increased in the narrower branches, cells reduced their minor axis and elongated, while simultaneously displacing the wall of the microtrack away from their cell body (Fig. 1e–g). Actin cytoskeleton remodeling and the actin polymerization required for force generation to displace the surrounding matrix both require cells to expend energy^15–17^, and we therefore hypothesized that cells require more energy to migrate in confined spaces.

### Confinement increases ATP:ADP ratio and glucose uptake

To measure changes in cellular energy state with spatial confinement, we used PercevalHR^31^, a ratiometric intracellular ATP:ADP fluorescent biosensor. ATP:ADP ratio is a sensitive indicator of changes in cellular energy^32^, and subcellular ATP:ADP gradients are essential for supporting energy-consuming processes underlying cell migration^33–35^. During migration, elevated ATP:ADP ratio correlates with increased glucose uptake and ATP hydrolysis, demonstrating the relationship between the biosensor signal and increased energy utilization^21^. Indeed, ATP:ADP ratio increased exponentially with spatial confinement in Y-shaped microtracks (Fig. 1h). As glucose is the primary source of cellular energy production and is correlated with cancer invasiveness^36^, we also examined changes in glucose uptake with spatial confinement. To measure glucose uptake, cells were incubated with the fluorescent glucose analog 2-NBDG. Consistently, 2-NBDG uptake increased with decreasing track width (Fig. 1i), indicating cells respond to confinement by increasing glucose uptake and energy production. Directly tracking intracellular ATP:ADP ratio during migration and decision-making through the Y-shaped microtrack showed that ATP:ADP ratio increased during migration from the feeder track into the branches, with higher ATP:ADP levels in the narrow branch compared with the wider branch (Fig. 1j, k; Supplementary Movies 3 and 4). Furthermore, we also assessed ATP:ADP ratio in tapered collagen microtracks that present a larger range of track width and observed a more pronounced increase in ATP:ADP ratio with confinement (Fig. 1l). Together, these results suggest that migration in narrower tracks requires more energy than migration in wider tracks, and migration decision-making may be influenced by the relative energetic costs between possible migration paths.

### Model of energy needs during confined migration

**Fig. 2:**
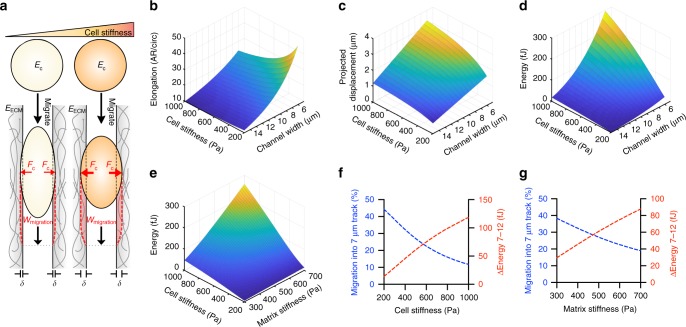
A model of energy requirements for confined migration explains cell decision-making. **a** The model of energy requirements for confined migration. A cell (with stiffness *E*_c_) migrating through a confined matrix (with stiffness *E*_ECM_) exerts force (*F*_c_) to displace the matrix (*δ*), requiring cellular work (*w*_migration_). **b**–**d** Model predictions for cell elongation (**b**), projected track displacement (**c**), and energetic requirements (**d**) for migration through confined spaces as a function of cell stiffness and channel width. **e** Model predictions for the energy landscape as a function of cell and collagen stiffness. **f**, **g** Predicted energetic costs between possible migration paths and probability of migration into the narrow path as a function of cell stiffness (**f**) and matrix stiffness (**g**)

Given the complexity of the integrated effects of cell and matrix mechanics on migration^1^, we created a computational model to probe cellular energy requirements for confined migration (Fig. 2a, Supplementary Table 1, see “Methods” section for details). We define the energy needed as the work that will be required to deform the cell/microtrack system when the cell moves by a normalized unit length. We only take into account the differences between the possible migration choices as the cell can only probe the choices provided and no future energy costs incurred after making the decision are known. An important assumption of the model is that the cells will actively try to adjust their shape to fit and spread within the microtrack, a process that will be a function of the overall stiffness of the system. Notably, while differences between cell speed would significantly alter energy use^21^ and thus the energetic cost, cell speed was found to be the same for all microtrack sizes (Supplementary Fig. 1). Therefore, the main difference in energetic costs between the microtracks will be from the work required to deform the collagen walls. We modeled the microtrack as two infinite half spaces (with stiffness *E*_ECM_) and a spread cell (with stiffness *E*_c_) as an elliptical soft body. We assume that cells larger than the width of the system uniformly exert force (*F*_c_) on each half space indenting the system (*δ*) depending on their size, shape, and compliance (Eq. (1)). Thus, the effective modulus of both the cell and microtrack will determine the amount of force exerted (Eq. (2)). To determine cell shape while maintaining constant perimeter, we imposed a limit to cell spreading in the microtrack based on cell stiffness and degree of spatial confinement (Fig. 2b, Eqs. (3) and (4)). The cell can now be described as an elliptical indenter, which will displace collagen side walls based on cell stiffness and confinement (Fig. 2c, Eq. (5)). Energy requirements for a cell moving within this confined space are then proportional to the work required to overcome the forces from the system deformation at equilibrium, which increases with cell stiffness, matrix stiffness, and confinement (Fig. 2d, e).

A probit model was used to estimate migration decision-making based on energetic costs. We assume that the standard deviation of the system is proportional to the minimal energy available for migration. Estimating the minimal energy required for migration as 0.19 pJ s^−1,^^37^, we calculated the probability of migration into the narrow path as a function of cell stiffness from the difference in energetic costs between migration paths (Fig. 2f, Eq. (6)). Similarly, we calculated the probability of migration into the narrow path as a function of ECM stiffness from the difference in energetic costs between migration paths (Fig. 2g, Eq. (6)). Our model predicts that cells preferentially migrate in the direction of energy minimalization, and stiffer cells or cells within stiffer ECM would be less likely to choose the narrower path. Together, this framework can be used to explain the role of energetics in decision-making during confined migration.

### Cell mechanics influence migration decision-making

**Fig. 3:**
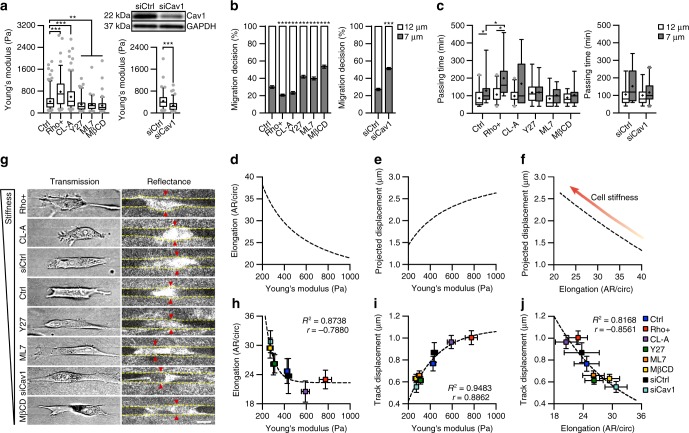
Cell stiffness alters decision-making and cell mechanics during confined migration. **a** AFM measurements of cell stiffness following pharmacological treatments and siCav1 knockdown (*n* = 164, 95, 167, 91, 75, 85, 92, and 97 cells for Ctrl, Rho+, CL-A, Y27, ML7, MβCD, siCtrl, and siCav1) and western blot for Caveolin-1 following treatment with 25 nM Caveolin-1 siRNA. **b**, **c** Migration decision-making (**b**) and passing time into each branch (**c**) following treatments to alter cell stiffness (*n* = 30, 34, 30, 31, 30, 28, 33, and 39 cells for Ctrl, Rho+, CL-A, Y27, ML7, MβCD, siCtrl, and siCav1). **d**–**f** Model predictions for cell deformation (**d**), track displacement (**e**), and the inverse relationship between cell elongation and track displacement (**f**) in 7 μm tracks. **g** Transmitted light images showing cell morphology and confocal reflectance images of microtrack structure as well as cell-induced track displacement in 7 μm microtracks (yellow lines show microtrack walls; red arrowheads show areas of matrix displacement around the cell body). **h**–**j** Experimental averages of cell elongation (**h**), track displacement (**i**), and the relationship between cell remodeling and matrix displacement (**j**) in 7 μm microtracks as a function of cell stiffness (*n* = 118, 107, 100, 135, 109, 123, 53, and 58 cells for Ctrl, Rho +, CL-A, Y27, ML7, MβCD, siCtrl, and siCav1). Data shown as median ± interquartile range (box), 5th–95th percentiles (whiskers), and mean (+) (**a**, **c**), or mean ± s.e.m. (**b**, **h**–**j**); dashed lines show one-phase decay (**h**, **j**) and one-phase association (**i**); two-tailed Mann–Whitney test (**a**, **c**) or two-tailed *t*-test (**b**); *P < 0.05, **P < 0.01, ***P < 0.001. Scale bar, 15 μm

We tested the robustness of our model by examining the influence of cell stiffness on migration decision-making. We manipulated MDA-MB-231 cell stiffness using pharmacological agents and short interfering RNA (siRNA)-mediated knockdown targeting cell contractility (Fig. 3a), since cell contractility and stiffness are an integrated system^38^. Treatment with a RhoA activator (Rho+) or Calyculin A (CL-A) increased cell stiffness, whereas treatment with Y27632 (Y27; a Rho-associated protein kinase inhibitor), ML7 (a myosin light chain kinase inhibitor), methyl-β-cyclodextrin (MβCD; a cholesterol depleting agent that causes actin disassembly^39^), or siRNA targeting Caveolin-1 (siCav1; a scaffolding protein of lipid rafts that influences actin remodeling^40^) decreased cell stiffness. Through these methods, we manipulated cell stiffness from ~271 to ~775 Pa (Fig. 3a). As predicted by the model, increased cell stiffness reduced migration into the narrower path, while decreased cell stiffness caused cells to become increasing agnostic to migration path (Fig. 3b). Notably, compliant cells were able to more readily pass into the narrow branch, while stiff cells required significantly more time at the bifurcation before passage (Fig. 3c).

We then evaluated how cell stiffness influenced cell and matrix remodeling during migration through confined spaces. Together, cell and matrix remodeling in combination with the width of the microtrack will determine the steric hindrance imposed on the cell body by the collagen matrix. Given our model assumes cell stiffness controls cell deformability^38^, increasing cell stiffness will result in a more rigid cytoskeleton that is more difficult to deform, and deformation of the collagen side walls will increase to fit the locomoting cell body (Fig. 3d–f). Note that these effects are expected to be most dramatic in tracks smaller than the cell body, where the matrix alone imposes high steric hindrance. These assumptions recapitulated cell stiffness-mediated changes in cell morphology and matrix deformation observed experimentally (Fig. 3g–j, Supplementary Fig. 2). Cell elongation, a measure of cell deformability, and cell-induced track displacement were inversely correlated in the 7 μm track, as cell elongation decreased with cell stiffness while track displacement increased with cell stiffness. These results validated the relationship between cell stiffness, cell deformation, and matrix remodeling defined in the model.

### Cell stiffness alters deformation to drive energetic costs

**Fig. 4:**
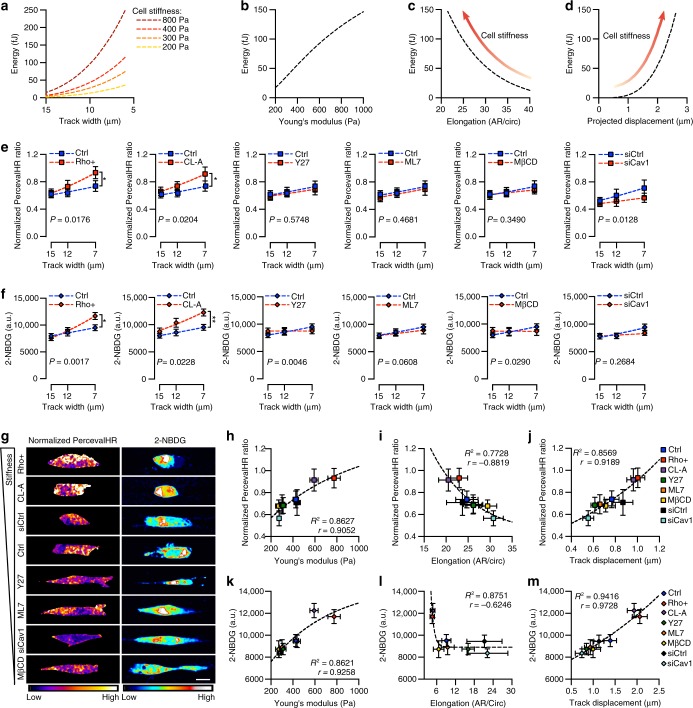
Cell stiffness-mediated increases in cellular ATP:ADP ratio and glucose uptake are correlated with matrix deformation. **a** Model predictions for energy requirements for migration into tracks of varying size with increasing cell stiffness. **b**–**d** Model predictions for energy requirements for migration (**b**), cell elongation (**c**), and track displacement (**d**) as a function of cell stiffness in 7 μm microtracks. **e** Normalized PercevalHR ratio with increasing confinement following pharmacological treatments and siCav1 knockdown (*n* = Ctrl: 111, 91, 118; Rho+: 111, 92, 107; CL-A: 121, 95, 100; Y27: 127, 117, 135; ML7: 114, 95, 107; MβCD: 116, 105, 123; siCtrl: 63, 49, 53; siCav1: 68, 46, 55 cells for 15, 12, 7 μm microtracks). **f** 2-NBDG uptake with increasing confinement following pharmacological treatments and siCav1 knockdown (*n* = Ctrl: 46, 29, 36; Rho+: 44, 41, 42; CL-A: 48, 40, 49; Y27: 61, 37, 46; ML7: 52, 38, 36; MβCD: 40, 24, 28; siCtrl: 46, 17, 22; siCav1: 46, 28, 21 cells for 15, 12, 7 μm microtracks). **g** Normalized PercevalHR ratio and 2-NBDG heatmap for cells of differing stiffness following treatment in 7 μm microtracks. **h**–**j** Normalized PercevalHR ratio as a function of cell stiffness (**h**), cell elongation (**i**), and track displacement (**j**) in 7 μm microtracks across cell stiffness altering treatments. **k**–**m** 2-NBDG uptake as a function of cell stiffness (**k**), cell elongation (**l**), and track displacement (**m**) in 7 μm microtracks across cell stiffness altering treatments. Data shown as mean ± s.e.m.; dashed lines show exponential growth (**e**, **f**, **j**, **m**); one-phase association (**h**, **k**), and one-phase decay (**i**, **l**); two-tailed Mann–Whitney test and extra sum-of-squares F-test for comparing curves (**e**, **f**); *P < 0.05, **P < 0.01. Scale bar, 15 μm

Our model predicts that increased energetic requirements in confined spaces are driven by increased force exerted on the matrix for displacement. Thus, energetic costs for migration are calculated to increase exponentially with confinement as a function of cell stiffness (Fig. 4a). Modeling migration through a 7 μm track predicts that energy requirements will exponentially increase with cell stiffness and cell-induced track displacement but decrease with cell deformability as cells become more compliant (Fig. 4b–d). Indeed, experimental results of intracellular ATP:ADP ratio and glucose uptake replicated model predictions, with ATP:ADP ratio and glucose uptake increasing with spatial confinement and amplifying in response to increased cell stiffness (Fig. 4e, f; Supplementary Fig. 3). In the 7 μm track, intracellular ATP:ATP ratio and glucose uptake increased as a function of cell stiffness (Fig. 4g). ATP:ADP ratio exponential increased with cell stiffness, and with increased cell stiffness, cell elongation negatively correlated with ATP:ADP ratio while track displacement strongly positively correlated with ATP:ADP ratio (Fig. 4h–j). Similarly, glucose uptake exponentially increased with cell stiffness and changes in glucose uptake were highly correlated with cell and matrix deformation (Fig. 4k–m). As expected, cell stiffness did not influence cellular energy levels in the feeder track and wider branch, where no significant cell-induced matrix displacement was observed (Supplementary Fig. 3). While cytoskeletal remodeling and matrix deformation both require cells to generate forces through increased ATP-dependent actin polymerization and actomyosin activity^15–17^, contractility inhibitors can affect ATP binding and Rho GTPase activity has been linked to cellular metabolism^41^. However, the close correlation between intracellular ATP:ADP ratio and track displacement as well as glucose uptake and track displacement indicate that the amount of force exerted on the matrix drives energetic costs for confined migration.

### Matrix stiffness alters decision-making and energetic costs

**Fig. 5:**
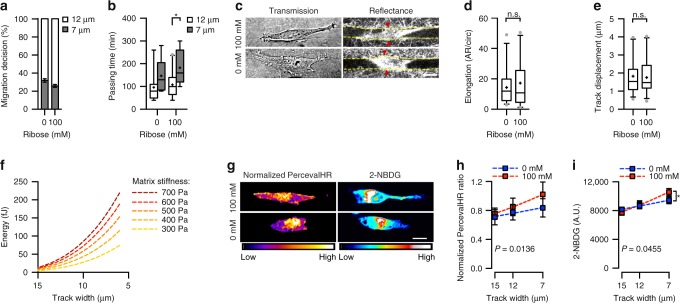
Matrix stiffness influences decision-making, cellular ATP:ADP ratio, and glucose uptake during confined migration. **a**, **b** Migration decision-making (**a**) and passing time into each branch (**b**) in 0 mM and 100 mM glycated collagen microtracks (*n* = 25, 27 cells for 0 mM, 100 mM). **c** Transmitted light images showing cell morphology and confocal reflectance images of microtrack structure as well as cell-induced track displacement in 7 μm microtracks (yellow lines show microtrack walls; red arrowheads show areas of matrix displacement around the cell body). **d**, **e** Quantification of cell elongation (**d**) and track displacement (**e**) for cells in the 7 μm track of 0 mM and 100 mM glycated collagen gels (*n* = 31, 29 cells for 0 mM, 100 mM). **f** Model predictions for energy requirements for migration into tracks of varying size with increasing cell stiffness. **g** Normalized PercevalHR heatmap and 2-NBDG heatmap of cells in matrices of varying stiffness following glycation. **h**, **i** Normalized PercevalHR ratio (*n* *=* 0 mM: 47, 30, 31; 100 mM: 49, 28, 29 cells for 15, 12, 7 μm tracks) (**h**) and 2-NBDG uptake (*n* *=* 0 mM: 96, 53, 59; 100 mM: 71, 46, 35 cells for 15, 12, 7 μm tracks) (**i**) with increasing confinement in collagen gels glycated with 0 mM or 100 mM ribose. Data shown as mean ± s.e.m. (**a**, **h**, **i**), or median ± interquartile range (box), 5th–95th percentiles (whiskers), and mean (+) (**b**, **d**, **e**); dashed lines show exponential fit; two-tailed Mann–Whitney test (**b**, **d**, **e**, **h**, **i**) and extra sum-of-squares F-test for comparing curves (**h**, **i**); *P < 0.05, n.s. = not significant. Scale bar, 15 μm

We further validated our model by altering matrix stiffness, as our model predicts force exerted on the system is determined by the effective modulus of both the cell and the collagen microtrack. This also allowed us to manipulate model parameters without directly altering cell behavior. To alter the matrix stiffness without changing matrix architecture, we utilized nonenzymatic glycation to form advanced glycation end product crosslinks^42^. Using 3.0 mg ml^−1^ collagen, increasing the extent of glycation from 0 to 100 mM increases the modulus of the matrix from ~400 to ~550 Pa^42^. As predicted, increased matrix stiffness decreased the propensity of cells to migrate into the narrower path and slowed passage time into the narrower path (Fig. 5a, b). No significant change was observed in cell elongation and matrix displacement with glycation (Fig. 5c–e), most likely due to relatively small range of stiffness evaluated. However, our model does calculate that energetic costs from migration will exponentially increase with confinement as a function of matrix stiffness (Fig. 5f). Importantly, we observed a larger increase in intracellular ATP:ADP levels and glucose uptake with increasing confinement for cells in collagen gels glycated with 100 mM ribose compared with cells in unglycated collagen tracks (Fig. 5g–i, Supplementary Fig. 4). These findings indicate that to achieve similar levels of matrix displacement during confined migration, cells in stiffer matrices must expend significantly more energy.

### Energetic costs are predictive of migration decision-making

**Fig. 6:**
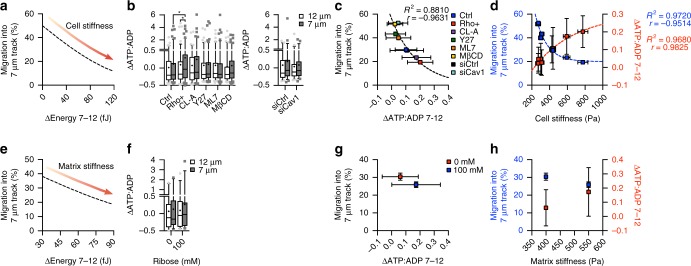
Energetic costs between migration choices govern migration decision-making. **a** Model predictions for migration choice as a function of the difference in energetic costs between migration paths with increasing cell stiffness. **b** ΔATP:ADP for migration into the 12 and 7 μm microtrack following pharmacological treatments and siCav1 knockdown (*n* = Ctrl: 91, 118; Rho+: 92, 107; CL-A: 95, 100; Y27: 117, 135; ML7: 95, 107; MβCD: 105, 123; siCtrl: 49, 53; siCav1: 46, 55 cells for 12 μm and 7 μm microtracks). **c** Percentage of migration into the 7 μm microtracks as a function of ΔATP:ADP 7–12 with changes in cell stiffness; dashed line shows one-phase decay. **d** The inverse relationship between migration frequency into the 7 μm microtracks and ΔATP:ADP 7–12 as a function of cell stiffness across treatments; dashed line shows one-phase decay for migration into 7 μm and one-phase association for ΔATP:ADP 7–12. **e** Model predictions for migration choice as function of energetic cost with increasing matrix stiffness. **f** ΔATP:ADP between migration paths with changing matrix stiffness (*n* = 0 mM: 30, 31; 100 mM: 31, 29 cells for 12 μm and 7 μm microtracks). **g** Percentage of migration into the 7 μm microtracks as a function of ΔATP:ADP 7–12 with changes in matrix stiffness. **h** The inverse relationship between migration frequency into the 7 μm microtracks and ΔATP:ADP 7–12 as a function of matrix stiffness. Data shown as median ± interquartile range (box), 5th–95th percentiles (whiskers), and mean (+) (**b**, **f**), or mean ± s.e.m. (**c**, **d**, **g**, **h**); two-tailed Mann–Whitney test (**b**, **f**); *P < 0.05

We then examined whether cell and matrix stiffness-mediated decision-making is governed by the difference in energetic costs between possible migration paths. Our model predicts the difference in energetic costs between the 7 and 12 μm track increases with cell stiffness, lowering the probability of migration into the narrow path (Fig. 6a). To test this, we measured energetic costs for migration as the difference in ATP:ADP ratio (ΔATP:ADP) between the feeder track and migration paths across experimental conditions (Fig. 6b). This allowed us to remove any changes in the ATP:ADP ratio due to pharmacological treatments and examine the energy differential between possible migration paths. ΔATP:ADP for migration into the 7 μm track was higher compared with ΔATP:ADP for migration into the 12 μm track for all treatments, and increased with cell stiffness (Fig. 6b). As predicted by the model (Fig. 2f), the difference in ATP:ADP ratio between the two migration paths (ΔATP:ADP 7–12) was inversely correlated with migration into the narrower track and exponentially increased with cell stiffness (Fig. 6c, d), indicating the lower ΔATP:ADP 7–12 of more compliant cells guided their more indiscriminate decision-making. Similarly, our model predicted that increasing matrix stiffness increases the energetic requirements for migration into the narrower track (Fig. 2g), lowering the probability of migration (Fig. 6e). We also found that ΔATP:ADP was higher in 7 μm track with elevated matrix stiffness (Fig. 6f) and ΔATP:ADP 7–12 was inversely correlated with migration into the narrow track with increased matrix stiffness (Fig. 6g, h). Hence, migration choice can be robustly predicted by assessing the difference in energetic costs for motility between possible migration paths.

## Discussion

The influence of the mechanical microenvironment on cell migration has been well studied;^1–3^ however, the energy needs of cells during migration, and how the mechanical microenvironment regulates energy needs, have largely been unexplored. Utilizing microfabrication techniques to recapitulate the architecture, composition, and mechanics of the in vivo ECM, we show that high physical confinement and steric hindrance inhibits migration due to elevated energetic requirements for cell-induced matrix displacement during migration. Notably, these increased energetic costs are determined by the mechanical properties of the cell and surrounding matrix, where high cell stiffness and/or matrix stiffness increase the cellular work necessary when migrating through confined spaces. We show that the energetic costs for motility between possible migration paths is predictive of the frequency of migration choice. Together, these findings provide a simple physical mechanism that links cellular energetics to cell mechanics and motility to explain migration decision-making in complex microenvironments.

Studying migration across multiple cancer cell lines has demonstrated that the physical properties of the cell correlate with a cell’s ability to migrate through confined environments^26^. In this study, our utilization of a single breast epithelial cancer cell line and modulating cell mechanical properties via contractility directly identifies cell stiffness as an important cell property for migration decision-making in confined spaces. We find more compliant cells need significantly less energy-intensive matrix remodeling for migration. Given cancer cells are frequently more compliant and deformable than healthy cells^43^ with cell compliance correlated with metastatic potential^44^, our results suggest that increased compliance may provide a phenotypic advantage to migrating cells as they require lower energetic costs for migration. Actomyosin-based activity expends a major portion of cellular energy^45^, and limiting the energy needed for cytoskeletal and matrix remodeling efforts during migration would be very advantageous to cancer cells. Deformability of cells is predominately regulated by the actin cytoskeleton^46^, and confinement can alter actin organization during both single cell^47^ and collective migration^48^. During confined migration, actin is redistributed to the cell poles and channel interfaces^7,47^. Compressive force acting on actin structures have been found to stimulate actin reorganization and promote the formation of a denser and overall stiffer actin network in vitro^49^. While this network stiffening is likely useful to push away the surrounding ECM to enable cell migration in microtracks as well as in other 3D migration systems^50^, it also increases energy consumption^49^. Indeed, we observed increased energy requirements in narrower tracks for stiffer, more rounded cells that would experience higher compressive forces from the matrix acting on their cell body. Thus, we propose that increased compliance allows cells to minimize energetic costs for migration through confined spaces and utilize more possible migration paths when navigating the stromal microenvironment. We demonstrate that cells are able to sense path size during migration and migration into narrower paths requires more time, indicating that increased probing and/or matrix and cell remodeling is necessary prior to passage. Understanding how cells are able to actively probe possible migration choices and identify the path of lowest energetic cost while navigating through the matrix will be an important challenge.

In addition to the mechanical properties of the cell, we show that the mechanical properties of the matrix also impose high energetic cost on migrating cells in physically constraining microtracks. Our model identifies that the effective modulus of both the cell and the collagen microtrack determine the force exerted on the system. Similar to stiff cells that are unable to elongate when moving into highly constricting tracks, cells migrating through narrow tracks in stiff matrices would experience increased mechanical loads on the cell body that increase actin network density and thus force^49^. Besides confinement and stiffness, other physical characteristics of the matrix including adhesion molecule expression also influence migration phenotype^2^. Physical confinement suppresses the formation of focal adhesions^47^ and cells under high confinement and low adhesion have been shown to undergo a switch from slow mesenchymal to fast ameboid-like migration^51^. However, the focal adhesion molecule vinculin maintains unidirectional migration in collagen microtracks^52^, and it has been suggested that forces transmitted by larger focal adhesions may function primarily to probe the matrix and guide in directional migration^53^. While changing collagen density, and therefore adhesion ligand expression, doesn’t alter migration speed in microtracks^7^, increasing matrix adhesivity may facilitate energy-intensive migration down a narrow path. Such changes in matrix properties would likely also lead to cytoskeletal changes, as the cytoskeleton serves as a mechanical coupler to the extracellular environment^54^. Taken together, these findings suggest a combination of both cell and matrix physical properties act to modulate cytoskeletal organization and dynamics during migration to drive energetic costs.

Recent work has linked metabolic alterations observed in cancer cells to energy-intensive cytoskeletal remodeling^55,56^. Most cancer cells rely on aerobic glycolysis instead of mitochondrial oxidative phosphorylation to meet energy needs, a phenomenon known as the Warburg effect^57^. Increased glycolytic activity is associated with a more aggressive phenotype^58,59^ to compensate for the enhanced ATP demand of cancer cells and rapidly produce energy^59,60^. The Warburg effect has been proposed as a metabolic strategy to optimally meet fluctuating energy demands and maintain functions inherent in an invasive malignant phenotype^20^. In migrating cancer cells, increased glycolytic activity is associated with greater cell motility and faster cytoskeletal remodeling, and ATP derived from glycolytic enzymes close to areas of active cytoskeletal rearrangement is critical for motility^55^. However, oxidative phosphorylation may also provide localized energy production to the most energy-demanding regions of the cell. Mitochondrial trafficking to the leading edge of the cell has been shown to be vital to cytoskeletal dynamics supporting membrane protrusion and focal adhesion dynamics necessary for cell migration^33,34^. Such localized energy production is also crucial for force generation and physical displacement of the matrix during MMP-independent migration. In matrices with high plasticity, cancer cells extend actin-rich invadopodia protrusions to physically widen channels in the matrix and facilitate protease-independent migration through confining microenvironments^50^. Similarly, during anchor cell invasion in *C. elegans*, mitochondria are trafficked to the invasive front delivering localized production of ATP for Arp2/3–F-actin network growth in large protrusions to physical breach and displace the basement membrane without MMPs^61^. Consistent with these findings, our observation that cellular ATP:ADP ratio and glucose uptake is highly correlated with cell-induced matrix displacement suggests that the energy production needed to drive cell-generated forces may drive confined migration. When passing through micrometric pores, rapid Arp2/3-nucleated perinuclear actin networks have also been shown to facilitate nuclear deformation and subsequent passage through constriction^62^. This mechanism can facilitate rapid migration through spatially complex and restricting microenvironments but may also require high levels of energy consumption.

The complex nature of cell migration presents challenges in therapeutically targeting migration, and endeavors to selectively inhibit cancer cell migration and metastasis have yielded limited success^63^. However, by linking cellular energetics to migration, the advent of new therapies targeting cancer metabolism may provide the foundation for treatments to target metastasis.

## Methods

### Cell culture and reagents

Highly metastatic MDA-MB-231 breast adenocarcinoma cells (HTB-26, ATCC) were maintained at 37 °C and 5% CO_2_ in Dulbecco’s Modified Eagle’s Medium (Life Technologies) supplemented with 10% fetal bovine serum (Atlanta Biologicals) and 1% penicillin–streptomycin (Life Technologies). For ATP:ADP studies, MDA-MB-231 cells were transduced with PercevalHR and pHRed as previously described^21^. Briefly, FUGW-PercevalHR (Addgene plasmid #49083) and GW1-pHRed (Addgene plasmid #31473) were gifts from Gary Yellen (Harvard Medical School, Boston, MA) and co-expressed in the MDA-MB-231 cell population. pFUW-CMV-pHRed was generated by inserting GW1-pHRed into the pFUW-CMV vector using *BamH1* and *EcoR1* restriction sites. Transient transfection of HEK293T (CRL-3216, ATCC) with lentiviral expression vectors and second-generation packing constructs psPAX2 and pMD2.G in TransIT-LT1 (Mirus) was performed, and lentiviral particles were harvested at 48 and 72 h post transfection. Lentiviral particles were then concentrated 100-fold with Lenti-X Concentrator (Clontech) and stably transduced into MDA-MB-231 cells in the presence of 8 μg ml^−1^ polybrene overnight (Santa Cruz Biotechnology). For studies manipulating cell stiffness using pharmacological agents targeting cell contractility, cells were treated with 0.125 μg ml^−1^ Rho Activator II (CN03, Cytoskeleton), 1 nM CL-A (Sigma-Aldrich), 10 μM Y27632 (VWR), 20 μM ML7 (EMD Millipore), 5 mM MβCD (Sigma-Aldrich), or their appropriate vehicle controls. All cell lines were tested and found negative for mycoplasma contamination.

### siRNA-mediated knockdown of Caveolin-1

MDA-MB-231 cells were transfected with 25–30 nM of scrambled control siRNA oligonucleotides (5′-UUCCUCUCCACGCGCAGUACAUUUA-3′), or 25–30 nM of Caveolin-1 siRNA oligonucleotides (5′-GGGACACACAGUUUUGACGUU-3′) using 2 μg ml^−1^ Lipofectamine 2000 (Invitrogen) in Opti-MEM transfection medium (Life Technologies). siRNA-mediated knockdown was confirmed by performing western blot 72 h post transfection. MDA-MB-231 cells transfected with siRNAs were lysed using preheated (at 90 °C) 2× Lammeli sample buffer after a quick rinse with ice-cold phosphate buffer saline (PBS) as described previously^64^. Briefly, cell lysates were subjected to sodium dodecyl sulfate-polyacrylamide gel electrophoresis with a Mini-PROTEAN Tetra System (Bio-Rad) and electro-transferred onto a polyvinylidene difluoride membrane. Blots were probed using polyclonal antibody against Caveolin-1 (PA1-064, Thermo Fisher Scientific) and glyceraldehyde-3-phosphate dehydrogenase (GAPDH; MAB374, Millipore). Anti-rabbit horseradish peroxidase conjugated secondary antibody (Rockland) was used against primary antibodies. After incubation with SuperSignal West Pico Chemiluminescent Substrate (Thermo Fisher Scientific), blots were exposed and imaged using a FujiFilm ImageQuant LAS-4000.

### Fabrication of collagen microtracks

Tapered and Y-shaped 3D collagen microtracks were prepared using micropatterning techniques. Photolithography was utilized to fabricate a 100 mm diameter silicon wafer mold consisting of an array of tapered wells with a 20–5 μm wide spatial gradient, and Y-shaped wells with a 15 μm wide lateral track bifurcating to 12 and 7 μm wide branches. End-to-end length of the tapered microtrack and the lateral track or branches of the Y-shaped microtrack were 1000 and 400 µm, respectively. All designs were created by L-Edit CAD software and transferred to chrome layered photomasks using a DWL2000 mask writer (Heidelberg Instruments). SU-8 25 negative photoresist (MicroChem) was spun to thickness of 25 µm on a silicon wafer, prebaked, and exposed to i-line UV-light (365 nm) using a contact aligner (ABM-USA, Inc.) equipped with a 350 nm long-pass filter. Following postexposure bake, the photoresist was developed using SU-8 developer (MicroChem) and treated with (1H,1H,2H,2H-Perfluorooctyl) Trichlorosilane as an antistiction coating. The silicon wafer mold was used to cast poly(dimethylsiloxane) (PDMS; Dow Corning) stamps by curing a ratio of 1:10 crosslinker to monomer at 60 °C for 2 h. Using the PDMS stamps, type I collagen isolated from rat tail tendons (Rockland Immunochemicals) was micromolded using a working collagen solution of 3.0 mg ml^−1^ from a 10 mg ml^−1^ collagen stock solution by diluting with ice-cold complete media and neutralizing the solution to pH 7.0 by adding 1 N NaOH, as described previously^27^. Collagen microtracks were prepared on plastic bottom six-well plates for phase-contrast imaging and no. 1.5 cover glass bottom six-well plates (Cellvis) were used for confocal imaging.

### Nonenzymatic glycation of collagen

As previously described^42^, 10 mg ml^−1^ collagen stock solutions were mixed with 0.5 M ribose to form solutions containing 0 or 100 mM ribose in 0.1% sterile acetic acid and incubated for 5 days at 4 °C. Glycated collagen solutions were then neutralized with 1N NaOH in 10× DPBS, HEPES (EMD Millipore) and sodium bicarbonate (J.T. Baker) to form 3.0 mg ml^−1^ collagen gels with 1× DPBS, 25 mM HEPES, and 44 mM sodium.

### Microtrack migration decision-making

For all 3D collagen microtrack migration experiments, cells were allowed to adhere for 6 h after seeding at a density of 70,000 cells ml^−1^. For cell migration decision-making studies in Y-shaped microtracks, all pharmacological agents were added with fresh complete media immediately prior to time-lapse imaging, except for Rho Activator II and MβCD, which were added with complete media after seeding. For MβCD treatment, seeded cells were incubated with MβCD for 4 h and then replaced with fresh complete cultured media prior imaging to avoid interference with cell viability^65,66^. All images were analyzed using ImageJ (version 2.0.0-rc-68/1.5g, National Institutes of Health). For cell migration decision-making studies, cells were carefully observed to determine their contact to one or two side walls of the track before reaching the bifurcation site. Cells that divided, interacted with other cells, or were blocked by other cells were excluded from the analysis. Time to decision was calculated as the time from when the cell body began interacting with the bifurcation of the Y-shaped microtrack in the feeder track to when the entire cell body was within the branch. For experiments assessing cell and matrix deformation, intracellular ATP:ADP ratio, and 2-NBDG uptake cells were allowed to migrate in the Y-shaped microtrack for at least 6 h following treatments as described above before measurements were taken.

### Phase-contrast microscopy

To study cell migration through collagen microtracks, time-lapse phase-contrast imaging was performed every 20 min for 12 h on a Zeiss Axio Observer Z1 inverted microscope equipped with a Hamamatsu ORCA-ER camera using a 10×/0.3 N.A. objective and operated by AxioVision software. Imaging was performed in an environmental chamber maintained at 37 °C and 5% CO_2_.

### Confocal microscopy

PercevalHR and pHRed signal as well as 2-NBDG uptake were imaged on a Zeiss LSM 800 inverted confocal microscope equipped with a 40×/1.1 N.A. long working distance water-immersion objective and operated by Zen 2.3 software. For measuring intracellular ATP:ADP ratio during time-lapse studies, a 20×/0.8 N.A. objective was used, and imaging was performed every 10 min for 12 h in an environmental chamber maintained at 37 °C and 5% CO_2_. PercevalHR was excited using a 488 and 405 nm laser corresponding to the ATP-bound and ADP-bound conformation, respectively^31^, and emission was collected through a 450–550 nm bandpass filter. pHRed was excited using a 561 and 488 nm laser and emission was collected through a 576 nm long-pass filter. 2-NBDG was excited using a 488 nm laser and emission was collected through a 490–650 nm bandpass filter. Cell morphology and collagen architecture was simultaneously imaged using the transmission and reflection channels, respectively.

### Confocal reflectance microscopy

Collagen architecture was visualized using a Zeiss LSM 800 inverted confocal microscope equipped with a 640 nm laser using a 40×/1.1 N.A. long working distance water-immersion objective and operated by Zen 2.3 software. Each collagen microtrack was visualized after fabrication. To account for changes in microtrack size during microtrack fabrication of Y-shaped tracks, only tracks within the following size parameters were used for this study: 15 μm track = 20–15 μm, 12 μm track = 11–13 μm, 7 μm track = <10 μm.

### Cell migration analysis

Cell velocity was measured by manually outlining cells in ImageJ and calculating the displacement of the cell centroid over time. Only cells tracked for more than 4 h were analyzed.

### Quantification of cell and matrix deformation

Cell features including minor axis, major axis, circularity, and aspect ratio were quantified using the measure tool in ImageJ after manually outlining the cell body. Elongation (aspect ratio/circularity) was calculated to assess change in cell shape and cell body deformation in the microtracks, as previously described^67^. Using confocal reflectance images, matrix deformation was calculated as the difference in microtrack width at the largest part of the cell body minus the microtrack width away from the cell body.

### Quantification of intracellular ATP:ADP ratio

Intracellular ATP:ADP ratio in MDA-MB-231 cells was calculated using PercevalHR and pHRed probes, as previously described^21,31^. Due to pH sensitivities of the PercevalHR sensor^31^, approximate removal of pH bias was performed using a pH calibration. Briefly, cells were treated with 15 mM NH_4_Cl to induce a transient alkalization of the cytosol and vary intracellular pH while maintaining an approximately constant ATP:ADP ratio. The pH calibration was performed over a short period of time (2–3 min) to minimize metabolic stress on cells and the linear correlation between uncorrected PercevalHR signal (F_488_/F_405_) and pHRed signal (F_561_/F_488_) was established to predict pH bias in PercevalHR signal. Only cells in the dynamic range of the linear correlation between uncorrected PercevalHR signal and pHRed signal were used in this study. PercevalHR signal was then normalized by dividing the uncorrected PercevalHR signal by the transformed pH-corrected signal.

Acquired PercevalHR and pHRed images were analyzed and normalized PercevalHR ratio was quantified in ImageJ using a customized macro. The mean background pixel intensity was measured and subtracted from the entire field of view for each channel to minimize interference from background noise. Using raw images, channels were then merged, subjected to a median filter (radius = 2 μm), and converted to a mask using a Li threshold. Pixels containing fluorescent signal were selected by applying the mask to background corrected images and the mean intensity for each channel was calculated, which were used to quantify the normalized PercevalHR ratio. To assess energetic costs during migration decision-making and account for any possible effects of pharmacological treatments, ΔATP:ADP was calculated as the ATP:ADP ratio of individual cells in the 12 or 7 μm track minus the average ATP:ADP ratio of cells in the 15 μm tracks. To assess energetic costs between the two possible migration paths, ΔATP:ADP 7–12 was calculated as the ATP:ADP ratio of individual cells in 7 μm track minus the average ATP:ADP ratio of cells in the 12 μm track.

### Quantification of glucose uptake

Glucose uptake was measured using fluorescent glucose analog 2-NBDG (Life Technologies), as previously described^21^ with some modifications. MDA-MB-231 cells were incubated in 0.146 mM 2-NBDG for 6 h, and then fixed with 3.2% paraformaldehyde (Sigma-Aldrich) in 1× PBS for 15 min at room temperature. Samples were then washed three times with 1× PBS for 15 min and then washed overnight in 1× PBS at 4 °C prior to imaging. To calculate 2-NBDG uptake, cells were manually outlined, and mean pixel intensity was calculated after background subtraction.

### Image generation

Representative images of intracellular ATP:ADP ratio, were generated as pixel-by-pixel ratio images and displayed as heatmaps using ImageJ. Adjustment of display map intensity, re-sizing, and addition of scale bars for all images was performed in ImageJ.

### Atomic force microscopy

AFM was performed using contact mode atomic force microscopy (MFP-3D, Asylum Research). For indentation testing, cells were plated on a collagen-coated glass and treated similarly with the pharmacological activators and inhibitors as mentioned for migration studies prior to probing with a silicon nitride cantilever having a nominal spring constant of 0.01 N m^−1^ and 4.5 μm diameter spherical polystyrene bead (Novascan). The spring constant of each probe was calibrated before each experiment and had a mean spring constant of 0.016 ± 0.004 N m^−1^. Force-displacement curves were obtained by indenting 1–3 locations on the cell periphery at a constant force of 500 nN and approach and retract speeds of 1 μm s^−1^. The Young’s modulus for each cell was determined by fitting force-displacement curves to the Hertz model assuming a Poisson’s ratio of 0.5 using the Asylum curve fitting software.

### Model of energetic costs for confined cell migration

To model migration through confined spaces, we first approximated our collagen microtracks as two infinite parallel half spaces with a given stiffness *E*_ECM_, determined by the surrounding matrix. For a 3.0 mg ml^−1^ collagen matrix, we assumed *E*_ECM_ to be 400 Pa^42^. In addition, the geometry of the experimental setup imposes a symmetry where only the width of the microtrack can be different, and the depth of all the microtrack is kept the same. This symmetry allows us to neglect the effect that would occur along the depth direction of the microtrack as any fluctuation would be minimal at best. To determine the cell size that will be used for the computation, we first approximated the cell as a spherical soft body. The average size, or diameter *D*_c_, of a cell in suspension is known to be ~18 μm for our MDA-MB-231 cellular model^68^. Thus, cells larger than the width of the system exert force *F*_c_ on each half space depending on their size and compliance *E*_c_. The governing force equation for one side of the parallel half space of this system for a cell with radius *R* and indentation *δ* is given by:

1 $$F_{\mathrm{c}} = \frac{4}{3}E_{{\mathrm{eff}}}\sqrt R \left( \delta \right)^{3/2}$$

The effective modulus of the cell and microtrack *E*_eff_ is given by:

2 $$E_{{\mathrm{eff}}} = \frac{{E_{\mathrm{c}}E_{{\mathrm{ECM}}}}}{{\left( {1 - \nu _{\mathrm{c}}^2} \right)E_{{\mathrm{ECM}}} + \left( {1 - \nu _{{\mathrm{ECM}}}^2} \right)E_{\mathrm{c}}}}$$

where *ν*_c_ is the Poisson’s ratio of the cell, and *ν*_ECM_ is the Poisson’s ratio of the matrix. One basic assumption is that the energy requirements for a soft body moving within this confined space should be proportional to the work required to overcome the forces from the system deformation at equilibrium. In this context, both cell shape and compliance will greatly influence the stress distribution. However, cell spreading within a confined space appears to be influenced by pathways controlling cell stiffness^69^. Interestingly, the data presented by Hung et al. suggests that cell spreading and elongation in a 6 μm wide confined space follows what appears to be an inverse exponential response as a function of stiffness^69^. To account for this cell feature, we can assume that a cell of stiffness *E*_c_ trying to fit within a channel of stiffness *E*_ECM_ will try to assume a shape of width *W*_c_ dependent on the overall stiffness of the system. Therefore, we can establish a relationship that links the apparent width the change in cell shape will impose to the stiffness of the system:

3 $$\frac{{dW_{\mathrm{c}}}}{{dE_{{\mathrm{eff}}}}} = - \gamma W_{\mathrm{c}}$$

Given the boundary conditions are fixed by the symmetry of the system and given the cell is interacting with the two walls, the new cell shape width parameter has to be within the limits *W*_track_ < *W*_c_ < *D*_c_, and the above equation solution can be reduced to:

4 $$W_{\mathrm{c}} = W_{{\mathrm{track}}} + \left( {D_{\mathrm{c}} - W_{{\mathrm{track}}}} \right)\left( {1 - e^{ - \gamma E_{{\mathrm{eff}}}}} \right)$$

where *W*_track_ is the width of the microtrack, and *γ* is the rate of change. 1/*γ* therefore represents the effective mean stiffness of the response. For the purpose of the model, we expect this parameter to be within the same range as the known Young’s modulus of the collagen scaffold. Using the computed cell height and assumed unspread cell diameter of 18 μm^68^, we can obtain the long axis of the ellipse that keeps the perimeter constant. The system can now be described as an elliptical indenter, which provides an effective contact radius *R* and indentation on each half space is:

5 $$\delta = (W_{\mathrm{c}} - W_{{\mathrm{track}}})/2$$

Of note, *δ* in the model corresponds to the apparent indentation as the real indentation depth of the deformed collagen wall and cell can only be solved numerically. Given the mechanical properties of the system, the model indicates that measurable deformation of the collagen side walls should increase with cell stiffness. The apparent indentation required to fit the cell body will then determine *F*_c_. Thus, the work for migration *w* is then defined as *F*_c_ multiplied by a normalized unit of movement of the cell through the microtrack. Furthermore, the model predicts that the energy difference between a 7 and 12 μm channel would be greater with stiffer cells. Therefore, our model indicates that the effective stiffness as well as the degree of spatial confinement directly impact the energy requirements for cells migrating in microtracks. Since there are only two possible migration choices in our experimental setup, a probit model can be utilized. A probit model is used to model binary outcome variables and has been widely applied as a standard method of reducing data to simple terms^70^. Therefore, we used a probit model to define the probabilistic outcome that would arise from the physical modeling results as the cumulative distribution of a standard normal function:

6 $$P\left( {t7{\mathrm{|}}t12,\sigma } \right) = \frac{1}{2}\left( {1 + erf\left[ {\frac{{w_{{\mathrm{t}}12} - w_{{\mathrm{t}}7}}}{{\sigma \sqrt 2 }}} \right]} \right)$$

In the current case, we can reasonably assume that the standard deviation of the system *σ* will be proportional to the energy available for cell migration. Using an estimate of 0.19 pJ s^−1^ for the minimal energy required for migration^37^, we can predict the probability of a cell choosing the smaller track as a function of its own stiffness or the stiffness of the surrounding ECM. A custom MATLAB (R2018a, Mathworks) code was used to generate the numerical results based on the model. All model parameters, their values, and their origins are described in Supplementary Table 1.

### Statistical analysis

All statistical analysis was performed using GraphPad Prism 7.0. Normality in the spread of data was tested using the D’Agostino–Pearson omnibus normality test. When two cases were compared, statistical significance was performed using a two-tailed Student’s *t*-test or a two-tailed Mann–Whitney test for data with non normal distribution. Multiple groups were compared using one-way ANOVA or Kruskal–Wallis test with Dunn’s post hoc analysis for data with non normal distribution. To determine significance in decision-making between wide and narrow migration paths, a one proportion calculation was performed and a Clopper–Pearson confidence interval for observed proportion was assessed. To determine if a curve adequately fit data or to compare two curves, the extra sum-of-squares F-test was used. Pearson’s correlation coefficient (r) was used to determine correlation. No statistical method was used to predetermine sample size. All experiments were reproduced at least three independent times.

### Reporting summary

Further information on research design is available in the Nature Research Reporting Summary linked to this article.

## Supplementary information

Supplementary Information

Reporting Summary

Description of Additional Supplementary Files

Supplementary Movie 1

Supplementary Movie 2

Supplementary Movie 3

Supplementary Movie 4

## Data Availability

A reporting summary for this article is available as a Supplementary Information file. The source data for this study are provided as a Source Data file.

## Source data

Source Data

## Code availability

The ImageJ macro (version 2.0.0-rc-68/1.5g, National Institutes of Health) used for quantification of normalized PercevalHR ratiometric signal and MATLAB code (R2018a, Mathworks) used to generate the computational model are available from the corresponding authors upon reasonable request.

## References

[1] Van Helvert S, Storm C, Friedl P (2018). Mechanoreciprocity in cell migration. Nat. Cell Biol..

[2] Paul CD, Mistriotis P, Konstantopoulos K (2017). Cancer cell motility: lessons from migration in confined spaces. Nat. Rev. Cancer.

[3] Charras G, Sahai E (2014). Physical influences of the extracellular environment on cell migration. Nat. Rev. Mol. Cell Biol..

[4] Lu P, Weaver VM, Werb Z (2012). The extracellular matrix: a dynamic niche in cancer progression. J. Cell Biol..

[5] Wolf K (2007). Multi-step pericellular proteolysis controls the transition from individual to collective cancer cell invasion. Nat. Cell Biol..

[6] Patsialou A (2013). Intravital multiphoton imaging reveals multicellular streaming as a crucial component of in vivo cell migration in human breast tumors. IntraVital.

[7] Carey SP (2015). Comparative mechanisms of cancer cell migration through 3D matrix and physiological microtracks. Am. J. Physiol. Cell Physiol..

[8] Doyle AD, Petrie RJ, Kutys ML, Yamada KM (2013). Dimensions in cell migration. Curr. Opin. Cell Biol..

[9] Wolf K (2009). Collagen-based cell migration models in vitro and in vivo. Semin. Cell Dev. Biol..

[10] Friedl P, Wolf K (2008). Tube travel: the role of proteases in individual and collective cancer cell invasion. Cancer Res..

[11] Coussens LM, Fingleton B, Matrisian LM (2002). Matrix metalloproteinase inhibitors and cancer: trials and tribulations. Science.

[12] Wyckoff JB, Pinner SE, Gschmeissner S, Condeelis JS, Sahai E (2006). ROCK- and myosin-dependent matrix deformation enables protease-independent tumor-cell invasion in vivo. Curr. Biol..

[13] Tozluoǧlu M (2013). Matrix geometry determines optimal cancer cell migration strategy and modulates response to interventions. Nat. Cell Biol..

[14] Wolf K, Friedl P (2011). Extracellular matrix determinants of proteolytic and non-proteolytic cell migration. Trends Cell Biol..

[15] Bursac P (2005). Cytoskeletal remodelling and slow dynamics in the living cell. Nat. Mater..

[16] Mizuno D, Tardin C, Schmidt CF, MacKintosh FC (2007). Nonequilibrium mechanics of active cytoskeletal networks. Science.

[17] Meshel AS, Wei Q, Adelstein RS, Sheetz MP (2005). Basic mechanism of three-dimensional collagen fibre transport by fibroblasts. Nat. Cell Biol..

[18] Balaban RS (1990). Regulation of oxidative phosphorylation in the mammalian cell. Am. J. Physiol. Cell Physiol..

[19] Epstein T, Xu L, Gillies RJ, Gatenby RA (2014). Separation of metabolic supply and demand: aerobic glycolysis as a normal physiological response to fluctuating energetic demands in the membrane. Cancer Metab..

[20] Epstein T, Gatenby RA, Brown JS (2017). The Warburg effect as an adaptation of cancer cells to rapid fluctuations in energy demand. PLoS One.

[21] Zanotelli MR (2018). Regulation of ATP utilization during metastatic cell migration by collagen architecture. Mol. Biol. Cell.

[22] Zhang J (2019). Energetic regulation of coordinated leader–follower dynamics during collective invasion of breast cancer cells. Proc. Natl Acad. Sci. USA.

[23] Mak M, Erickson D (2014). Mechanical decision trees for investigating and modulating single-cell cancer invasion dynamics. Lab Chip.

[24] Ambravaneswaran V, Wong IY, Aranyosi AJ, Toner M, Irimia D (2010). Directional decisions during neutrophil chemotaxis inside bifurcating channels. Integr. Biol..

[25] Paul CD (2016). Interplay of the physical microenvironment, contact guidance, and intracellular signaling in cell decision making. FASEB J..

[26] Lautscham LA (2015). Migration in confined 3D environments Is determined by a combination of adhesiveness, nuclear volume, contractility, and cell stiffness. Biophys. J..

[27] Kraning-Rush CM, Carey SP, Lampi MC, Reinhart-King CA (2013). Microfabricated collagen tracks facilitate single cell metastatic invasion in 3D. Integr. Biol..

[28] TruongVo T N, Kennedy R M, Chen H, Chen A, Berndt A, Agarwal M, Zhu L, Nakshatri H, Wallace J, Na S, Yokota H, Ryu J E (2017). Microfluidic channel for characterizing normal and breast cancer cells. Journal of Micromechanics and Microengineering.

[29] Fu Y, Chin LK, Bourouina T, Liu AQ, Vandongen AMJJ (2012). Nuclear deformation during breast cancer cell transmigration. Lab Chip.

[30] Wolf K (2013). Physical limits of cell migration: control by ECM space and nuclear deformation and tuning by proteolysis and traction force. J. Cell Biol..

[31] Tantama M (2013). Imaging energy status in live cells with a fluorescent biosensor of the intracellular ATP-to-ADP ratio. Nat. Commun..

[32] Yuan HX, Xiong Y, Guan KL (2013). Nutrient sensing, metabolism, and cell growth control. Mol. Cell.

[33] Cunniff B, McKenzie AJ, Heintz NH, Howe AK (2016). AMPK activity regulates trafficking of mitochondria to the leading edge during cell migration and matrix invasion. Mol. Biol. Cell.

[34] Schuler M-H (2017). Miro1-mediated mitochondrial positioning shapes intracellular energy gradients required for cell migration. Mol. Biol. Cell.

[35] Van Horssen R (2009). Modulation of cell motility by spatial repositioning of enzymatic ATP/ADP exchange capacity. J. Biol. Chem..

[36] Gillies RJ, Robey I, Gatenby RA (2008). Causes and consequences of increased glucose metabolism of cancers. J. Nucl. Med..

[37] Hecht I (2015). The motility-proliferation-metabolism interplay during metastatic invasion. Sci. Rep..

[38] Wang N, Ingber DE (1994). Control of cytoskeletal mechanics by extracellular matrix, cell shape, and mechanical tension. Biophys. J..

[39] Chubinskiy-Nadezhdin VI, Efremova TN, Khaitlina SY, Morachevskaya EA (2013). Functional impact of cholesterol sequestration on actin cytoskeleton in normal and transformed fibroblasts. Cell Biol. Int..

[40] Echarri A, Del Pozo MA (2015). Caveolae - mechanosensitive membrane invaginations linked to actin filaments. J. Cell Sci..

[41] Wang JBin (2010). Targeting mitochondrial glutaminase activity inhibits oncogenic transformation. Cancer Cell.

[42] Bordeleau F (2016). Matrix stiffening promotes a tumor vasculature phenotype. Proc. Natl Acad. Sci. USA.

[43] Cross SE, Yu-Sheng J, Jianyu R, Gimzewski JK (2007). Nanomechanical analysis of cells from cancer patients. Nat. Nanotechnol..

[44] Guck J (2005). Optical deformability as an inherent cell marker for testing malignant transformation and metastatic competence. Biophys. J..

[45] Bernstein BW, Bamburg JR (2003). Actin-ATP hydrolysis is a major energy drain for neurons. J. Neurosci..

[46] Ananthakrishnan R (2006). Quantifying the contribution of actin networks to the elastic strength of fibroblasts. J. Theor. Biol..

[47] Balzer EM (2012). Physical confinement alters tumor cell adhesion and migration phenotypes. FASEB J..

[48] Xi W, Sonam S, Beng Saw T, Ladoux B, Teck Lim C (2017). Emergent patterns of collective cell migration under tubular confinement. Nat. Commun..

[49] Bieling P (2016). Force feedback controls motor activity and mechanical properties of self-assembling branched actin networks. Cell.

[50] Wisdom KM (2018). Matrix mechanical plasticity regulates cancer cell migration through confining microenvironments. Nat. Commun..

[51] Liu YJ (2015). Confinement and low adhesion induce fast amoeboid migration of slow mesenchymal cells. Cell.

[52] Rahman A (2016). Vinculin regulates directionality and cell polarity in two- and three-dimensional matrix and three-dimensional microtrack migration. Mol. Biol. Cell.

[53] Bergert M (2015). Force transmission during adhesion-independent migration. Nat. Cell Biol..

[54] Zanotelli MR, Bordeleau F, Reinhart-King CA (2017). Subcellular regulation of cancer cell mechanics. Curr. Opin. Biomed. Eng..

[55] Shiraishi T (2015). Glycolysis is the primary bioenergetic pathway for cell motility and cytoskeletal remodeling in human prostate and breast cancer cells. Oncotarget.

[56] Hu H (2016). Phosphoinositide 3-kinase regulates glycolysis through mobilization of aldolase from the actin cytoskeleton. Cell.

[57] Vander Heiden MG, Cantley LC, Thompson CB (2009). Understanding the Warburg effect: the metabolic requirements of cell proliferation. Science.

[58] Postovit LM, Adams MA, Lash GE, Heaton JP, Graham CH (2002). Oxygen-mediated regulation of tumor cell invasiveness: involvement of a nitric oxide signaling pathway. J. Biol. Chem..

[59] Gatenby RA, Gillies RJ (2004). Why do cancers have high aerobic glycolysis?. Nat. Rev. Cancer.

[60] Lunt SY, Vander Heiden MG (2011). Aerobic glycolysis: meeting the metabolic requirements of cell proliferation. Annu. Rev. Cell Dev. Biol..

[61] Kelley LC (2019). Adapative F-actin polymerization and localized ATP production drive basement membrane invasion in the absence of MMPs. Dev. Cell.

[62] Thiam HR (2016). Perinuclear Arp2/3-driven actin polymerization enables nuclear deformation to facilitate cell migration through complex environments. Nat. Commun..

[63] Steeg PS (2006). Tumor metastasis: mechanistic insights and clinical challenges. Nat. Med..

[64] Huynh J, Bordeleau F, Kraning-Rush CM, Reinhart-King CA (2013). Substrate stiffness regulates PDGF-induced circular dorsal ruffle formation through MLCK. Cell. Mol. Bioeng..

[65] Guerra FS (2016). Membrane cholesterol depletion reduces breast tumor cell migration by a mechanism that involves non-canonical Wnt signaling and IL-10 secretion. Transl. Med. Commun..

[66] Yang YT (2012). Characterization of cholesterol-depleted or -restored cell membranes by depth-sensing nano-indentation. Soft Matter.

[67] Carey SP (2016). Local extracellular matrix alignment directs cellular protrusion dynamics and migration through Rac1 and FAK. Integr. Biol..

[68] Kim U (2007). Selection of mammalian cells based on their cell-cycle phase using dielectrophoresis. Proc. Natl Acad. Sci. USA.

[69] Hung WC (2013). Distinct signaling mechanisms regulate migration in unconfined versus confined spaces. J. Cell Biol..

[70] Finney, D. J. *Probit Analysis: A Statistical Treatment of the Sigmoid Response Curve*. (Cambridge university press, 1962).

