# Author Correction: Energetic costs regulated by cell mechanics and confinement are predictive of migration path during decision-making

**DOI:** 10.1038/s41467-019-13655-8

**Published:** 2019-12-04

**Authors:** Matthew R. Zanotelli, Aniqua Rahman-Zaman, Jacob A. VanderBurgh, Paul V. Taufalele, Aadhar Jain, David Erickson, Francois Bordeleau, Cynthia A. Reinhart-King

**Affiliations:** 1 Nancy E. and Peter C. Meinig School of Biomedical Engineering, Cornell University, Ithaca, NY 14853 USA; 2 Department of Biomedical Engineering, Vanderbilt University, Nashville, TN 37235 USA; 3 Sibley School of Mechanical and Aerospace Engineering, Cornell University, Ithaca, NY 14853 USA; 4 CHU de Québec-Université Laval Research Center (Oncology division), Université Laval Cancer Research Center and Faculty of Medicine, Université Laval, Québec, QC G1R 3S3 Canada

**Keywords:** Extracellular matrix, Biomedical engineering

Correction to: *Nature Communications* 10.1038/s41467-019-12155-z, published online 13 September 2019.

The original version of this Article contained an error in Fig. 4, in which Fig. 4c and Fig. 4d were inadvertently switched. This has now been corrected in the PDF and HTML versions of the Article.

