# Supplementary material for: Energetic costs regulated by cell mechanics and confinement are predictive of migration path during decision-making
Source: Nat Commun. 2019 Sep 13;10:4185. doi: 10.1038/s41467-019-12155-z (PMC6744572; doi:10.1038/s41467-019-12155-z)
Supplement: Supplementary file 1 — Supplementary Information [file 41467_2019_12155_MOESM1_ESM.pdf]

Supplementary Information

Energetic costs regulated by cell mechanics and confinement are predictive of migration path during decision-making

Zanotelli et al.

Supplementary Table 1. Model parameters.

Parameter	Meaning	Value	Origin
D_c	Assumed normal cell diameter	18 μm	[1]
E_c	Cell stiffness	200-1000 Pa	Adjusted, based on values measured here
ν_c	Poisson's ratio of the cell	0.45	Assumed
E_{ECM}	Extracellular matrix stiffness	400-550 Pa	[2]
ν_{ECM}	Poisson's ratio of the extracellular matrix	0.45	Assumed
σ	Minimal energy required for migration	0.19 pJ s^{-1}	[3]

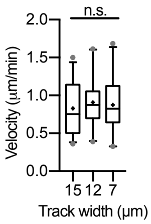

Supplementary Figure 1. Cell migration speed and spatial confinement. Quantification of cell velocity with increasing confinement (*n* = 34, 33, 22 cells for 15, 12, 7 µm tracks). Data shown as median ± interquartile range (box), 5th–95th percentiles (whiskers), and mean (+); Kruskal-Wallis test with Dunn's post hoc analysis; n.s. = not significant.

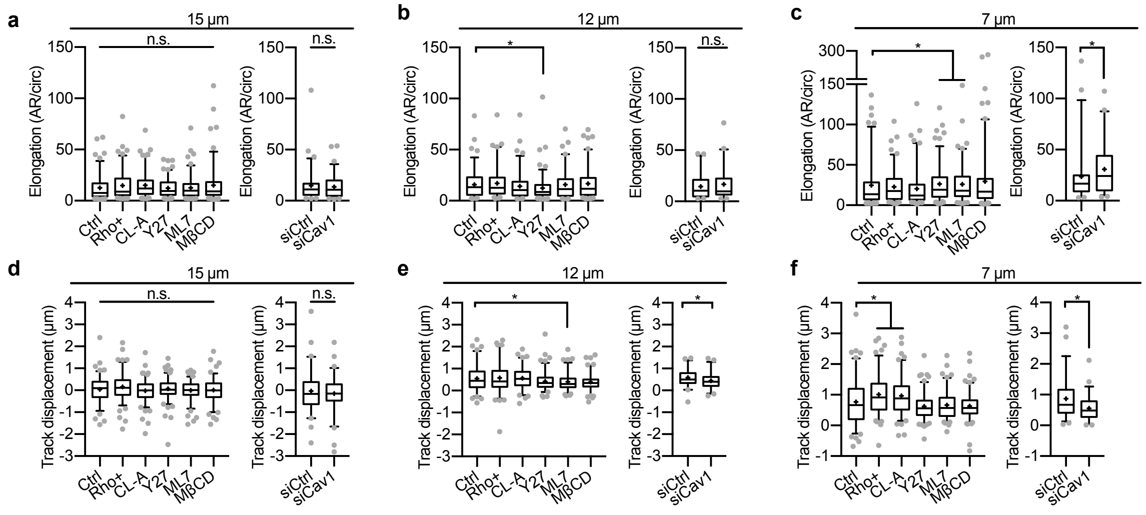

Supplementary Figure 2. Cell stiffness-mediated changes in cell and matrix deformation increase with spatial confinement. a–c, Quantification of cell elongation following pharmacological treatments and siCav1 knockdown in 15 μm (**a**), 12 μm (**b**), and 7 μm (**c**) collagen microtracks. **d–f**, Quantification of track deformation post pharmacological treatments and siCav1 knockdown in 15 μm (**d**), 12 μm (**e**), and 7 μm (**f**) collagen microtracks (*n* = Ctrl: 111, 91, 118; Rho+: 111, 92, 107; CL-A: 121, 95, 100; Y27: 127, 117, 135; ML7: 114, 95, 107; MβCD: 116, 105, 123; siCtrl: 63, 49, 53; siCav1: 68, 46, 55 cells for 15, 12, 7 μm microtracks). Data shown as median ± interquartile range (box), 5th–95th percentiles (whiskers), and mean (+); two-tailed Mann–Whitney test; *P<0.05, n.s. = not significant.

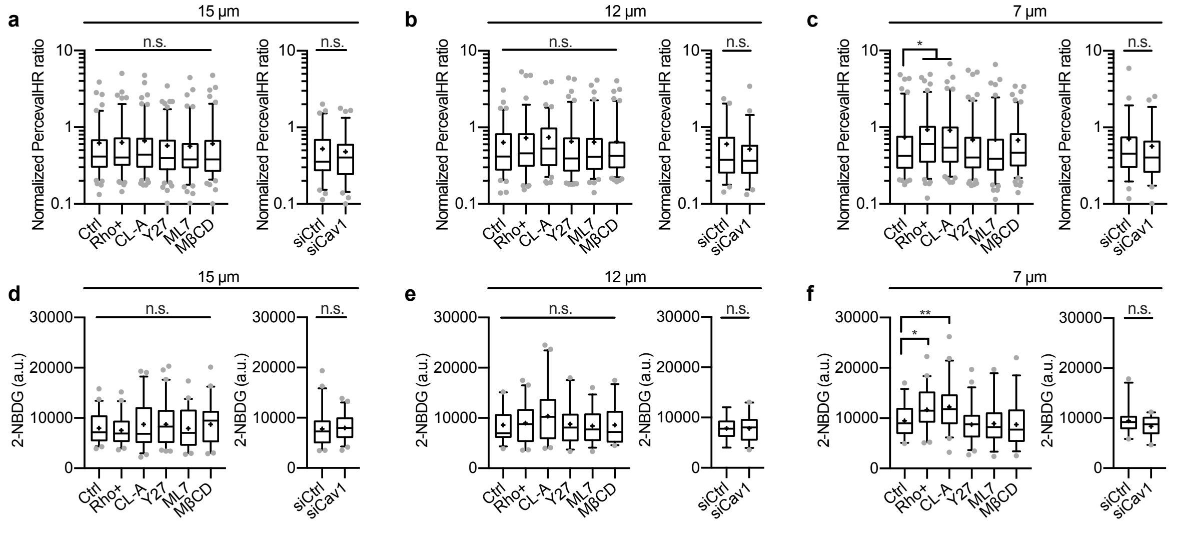

Supplementary Figure 3. Cell stiffness-mediated changes in cellular ATP:ADP ratio and glucose uptake increase with spatial confinement. a–c, Quantification of normalized PercevalHR ratio following pharmacological treatments and siCav1 knockdown in 15 µm (**a**), 12 µm (**b**), and 7 µm (**c**) collagen microtracks (*n* = Ctrl: 111, 91, 118; Rho+: 111, 92, 107; CL-A: 121, 95, 100; Y27: 127, 117, 135; ML7: 114, 95, 107; MβCD: 116, 105, 123; siCtrl: 63, 49, 53; siCav1: 68, 46, 55 cells for 15, 12, 7 µm microtracks). **d–f**, Quantification of 2-NBDG uptake following pharmacological treatments and siCav1 knockdown in 15 µm (**d**), 12 µm (**e**), and 7 µm (**f**) collagen microtracks (*n* = Ctrl: 46, 29, 36; Rho+: 44, 41, 42; CL-A:48, 40, 49; Y27:61, 37, 46; ML7: 52, 38, 36; MβCD: 40, 24, 28; siCtrl: 46, 17, 22; siCav1: 46, 28, 21 cells for 15, 12, 7 µm microtracks). Data shown as median ± interquartile range (box), 5th–95th percentiles (whiskers), and mean (+); two-tailed Mann–Whitney test; **P*<0.05, ***P*<0.01, n.s. = not significant.

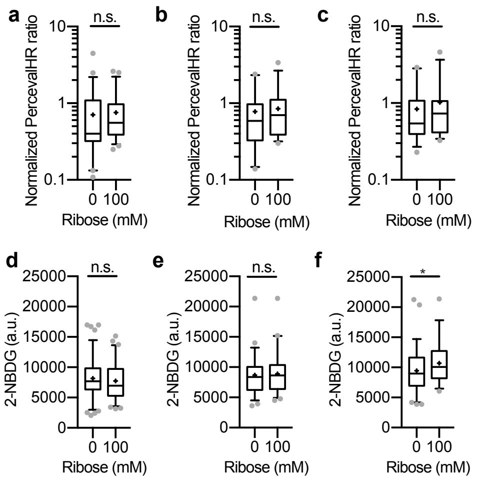

Supplementary Figure 4. Matrix stiffness-mediated changes in cellular ATP:ADP ratio and glucose uptake with spatial confinement. a–**c**, Quantification of normalized PercevalHR ratio for 15 µm (**a**), 12 µm (**b**), and 7 µm (**c**) microtracks in collagen gels glycated with 0 mM or 100 mM ribose (*n* = 0 mM: 47, 30, 31; 100 mM: 49, 28, 29 cells for 15, 12, 7 µm tracks). **d**–**f**, Quantification of 2-NBDG uptake in 15 µm (**d**), 12 µm (**e**), and 7 µm (**f**) microtracks in collagen gels glycated with 0 mM or 100 mM ribose (*n* = 0 mM: 96, 53, 59; 100 mM: 71, 46, 35 cells for 15, 12, 7 µm tracks). Data shown as median ± interquartile range (box), 5th–95th percentiles (whiskers), and mean (+); two-tailed Mann–Whitney test; *P<0.05, n.s. = not significant.

Supplementary References

1. Kim, U. *et al.* Selection of mammalian cells based on their cell-cycle phase using dielectrophoresis. *Proc. Natl. Acad. Sci.* **104**, 20708–12 (2007).
2. Bordeleau, F. *et al.* Matrix stiffening promotes a tumor vasculature phenotype. *Proc. Natl. Acad. Sci.* **114**, 492–497 (2016).
3. Hecht, I. *et al.* The motility-proliferation-metabolism interplay during metastatic invasion. *Sci. Rep.* **5**, 13538 (2015).
