# Supplementary material for: Energetic costs regulated by cell mechanics and confinement are predictive of migration path during decision-making
Source: Nat Commun. 2019 Sep 13;10:4185. doi: 10.1038/s41467-019-12155-z (PMC6744572; doi:10.1038/s41467-019-12155-z)
Supplement: Supplementary file 2 — Reporting Summary [file 41467_2019_12155_MOESM2_ESM.pdf]

nature research

Corresponding author(s): Cynthia A. Reinhart-King

Reporting Summary

Nature Research wishes to improve the reproducibility of the work that we publish. This form provides structure for consistency and transparency in reporting. For further information on Nature Research policies, see Authors & Referees and the Editorial Policy Checklist.

Statistical parameters

When statistical analyses are reported, confirm that the following items are present in the relevant location (e.g. figure legend, table legend, main text, or Methods section).

n/a	Confirmed	
☐	☒	The exact sample size (*n*) for each experimental group/condition, given as a discrete number and unit of measurement
☐	☒	An indication of whether measurements were taken from distinct samples or whether the same sample was measured repeatedly
☐	☒	The statistical test(s) used AND whether they are one- or two-sided<br>*Only common tests should be described solely by name; describe more complex techniques in the Methods section.*
☐	☒	A description of all covariates tested
☐	☒	A description of any assumptions or corrections, such as tests of normality and adjustment for multiple comparisons
☐	☒	A full description of the statistics including central tendency (e.g. means) or other basic estimates (e.g. regression coefficient) AND variation (e.g. standard deviation) or associated estimates of uncertainty (e.g. confidence intervals)
☐	☒	For null hypothesis testing, the test statistic (e.g. *F*, *t*, *r*) with confidence intervals, effect sizes, degrees of freedom and *P* value noted<br>*Give P values as exact values whenever suitable.*
☒	☐	For Bayesian analysis, information on the choice of priors and Markov chain Monte Carlo settings
☒	☐	For hierarchical and complex designs, identification of the appropriate level for tests and full reporting of outcomes
☐	☒	Estimates of effect sizes (e.g. Cohen's *d*, Pearson's *r*), indicating how they were calculated
☐	☒	Clearly defined error bars<br>*State explicitly what error bars represent (e.g. SD, SE, CI)*

Our web collection on statistics for biologists may be useful.

Software and code

Policy information about availability of computer code

Data collection	Software used for data collection included Zen 2.3 for confocal microscopy, AxioVision v.4.8.10 for phase contrast microscopy, and Igor Pro 6.27 for atomic force microscopy.
Data analysis	All data analysis was performed in ImageJ 2.0.0. PercevalHR signal and pHRed signal used to calculate normalized PercevalHR ratio were analyzed using a custom macro in ImageJ 2.0.0. Numerical results based on the physical model were calculated from custom code in MATLAB R2018a. Averaging was performed in Excel and GraphPad Prism 7. Statistical analysis was performed in GraphPad Prism 7.

For manuscripts utilizing custom algorithms or software that are central to the research but not yet described in published literature, software must be made available to editors/reviewers upon request. We strongly encourage code deposition in a community repository (e.g. GitHub). See the Nature Research guidelines for submitting code & software for further information.

Data

Policy information about availability of data

All manuscripts must include a data availability statement. This statement should provide the following information, where applicable:

- Accession codes, unique identifiers, or web links for publicly available datasets
- A list of figures that have associated raw data
- A description of any restrictions on data availability

The source data for this study are provided as a Source Data file. Custom software used for analysis are available from the corresponding author upon reasonable request.

Field-specific reporting

Please select the best fit for your research. If you are not sure, read the appropriate sections before making your selection.

☒ Life sciences ☐ Behavioural & social sciences ☐ Ecological, evolutionary & environmental sciences

For a reference copy of the document with all sections, see nature.com/authors/policies/ReportingSummary-flat.pdf

Life sciences study design

All studies must disclose on these points even when the disclosure is negative.

Sample size	No statistical method was used to predetermine sample size.
Data exclusions	Cells that divided, interacted with other cells, or were blocked by other cells were excluded from analysis. To account for changes in microtrack size during microtrack fabrication of Y-shaped tracks, only tracks within the following size parameters were used: 15 µm track = 20-15 µm, 12 µm track = 11-13 µm, 7 µm track = <10 µm. For ATP:ADP analysis, only cells in the dynamic range of the linear correlation between uncorrected PercevalHR and pHRed signal were used in this study.
Replication	All experimental findings were reproduced independently at least three times.
Randomization	Cells were measured at random within each condition.
Blinding	Investigators were not blinded to group allocation.

Reporting for specific materials, systems and methods

Materials & experimental systems

n/a	Involved in the study
☐	☒ Unique biological materials
☐	☒ Antibodies
☐	☒ Eukaryotic cell lines
☒	☐ Palaeontology
☒	☐ Animals and other organisms
☒	☐ Human research participants

Methods

n/a	Involved in the study
☒	☐ ChIP-seq
☒	☐ Flow cytometry
☒	☐ MRI-based neuroimaging

Unique biological materials

Policy information about availability of materials

Obtaining unique materials	All collagen matrices were prepared using type I collagen extracted from rat tail tendons commercially available from Rockland Immunochemicals.

Antibodies

Antibodies used	anti-Caveolin-1 (PA1-064, Thermo Fisher Scientific); anti-GAPDH (MAB374, Millipore); anti-HRP conjugated secondary (Rockland).

Validation	Antibodies were validated by the manufacturer prior to purchasing.

Eukaryotic cell lines

Policy information about cell lines

Cell line source(s)	Highly metastatic MDA-MB-231 breast adenocarcinoma cells (HTB-26) and HEK-293T cells (CRL-3216) were purchased from ATCC.
Authentication	All cell lines were originally authenticated by ATCC and were not further authenticated.
Mycoplasma contamination	All cell lines were tested and found negative for mycoplasma contamination.
Commonly misidentified lines (See ICLAC register)	HEK-293T cells were used for lentiviral production.
