# Supplementary material for: Energetic costs regulated by cell mechanics and confinement are predictive of migration path during decision-making
Source: Nat Commun. 2019 Sep 13;10:4185. doi: 10.1038/s41467-019-12155-z (PMC6744572; doi:10.1038/s41467-019-12155-z)
Supplement: Supplementary file 3 — Description of Additional Supplementary Files [file 41467_2019_12155_MOESM3_ESM.pdf]

Description of Additional Supplementary Files

Title: Supplementary Movie 1. Cell migration decision-making into the wider path.
Description: Phase contrast time-lapse imaging of MDA-MB-231 cell migration through the 15 μm feeder track into the 12 μm branch of a Y-shaped collagen microtrack. Time in h:min. Scale bar, 20 μm.

Title: Supplementary Movie 2. Cell migration decision-making into the narrower path.
Description: Phase contrast time-lapse imaging of MDA-MB-231 cell migration through the 15 μm feeder track into the 7 μm branch of a Y-shaped collagen microtrack. Time in h:min. Scale bar, 20 μm.

Title: Supplementary Movie 3. Intracellular ATP:ADP ratio during migration into the wider path.
Description: Confocal fluorescent time-lapse imaging of the normalized PercevalHR ratio (intracellular ATP:ADP ratio) of MDA-MB-231 cell migration through the 15 μm feeder track into the 12 μm branch of a Y-shaped collagen microtrack. Intracellular ATP:ADP levels were assigned a hue on the color spectrum from purple (low intracellular ATP:ADP ratio) to yellow (high intracellular ATP:ADP ratio). Time in h:min. Scale bar, 20 μm.

Title: Supplementary Movie 4. Intracellular ATP:ADP ratio during migration into the narrow path.
Description: Confocal fluorescent time-lapse imaging of the normalized PercevalHR ratio (intracellular ATP:ADP ratio) of MDA-MB-231 cell migration through the 15 μm feeder track into the 7 μm branch of a Y-shaped collagen microtrack. Intracellular ATP:ADP levels were assigned a hue on the color spectrum from purple (low intracellular ATP:ADP ratio) to yellow (high intracellular ATP:ADP ratio). Time in h:min. Scale bar, 20 μm.
